# Three-dimensional microCT imaging of murine embryonic development from immediate post-implantation to organogenesis: application for phenotyping analysis of early embryonic lethality in mutant animals

**DOI:** 10.1007/s00335-017-9723-6

**Published:** 2017-11-23

**Authors:** Olga Ermakova, Tiziana Orsini, Alessia Gambadoro, Francesco Chiani, Glauco P. Tocchini-Valentini

**Affiliations:** 1 Institute of Cell Biology and Neurobiology (IBCN), via Ramarini, 32, 00015 Monterotondo, Rome, Italy; 2 European Mouse Mutant Archive (EMMA), Monterotondo Mouse Clinic, via Ramarini, 32, 00015 Monterotondo, Rome, Italy

## Abstract

In this work, we applied three-dimensional microCT imaging to study murine embryogenesis in the range from immediate post-implantation period (embryonic day 5.5) to mid-gestation (embryonic day 12.5) with the resolution up to 1.4 µm/voxel. Also, we introduce an imaging procedure for non-invasive volumetric estimation of an entire litter of embryos within the maternal uterine structures. This method allows for an accurate, detailed and systematic morphometric analysis of both embryonic and extra-embryonic components during embryogenesis. Three-dimensional imaging of unperturbed embryos was performed to visualize the egg cylinder, primitive streak, gastrulation and early organogenesis stages of murine development in the C57Bl6/N mouse reference strain. Further, we applied our microCT imaging protocol to determine the earliest point when embryonic development is arrested in a mouse line with knockout for tRNA splicing endonuclease subunit *Tsen54* gene. Our analysis determined that the embryonic development in *Tsen54* null embryos does not proceed beyond implantation. We demonstrated that application of microCT imaging to entire litter of non-perturbed embryos greatly facilitate studies to unravel gene function during early embryogenesis and to determine the precise point at which embryonic development is arrested in mutant animals. The described method is inexpensive, does not require lengthy embryos dissection and can be applicable for detailed analysis of mutant mice at laboratory scale as well as for high-throughput projects.

**Electronic supplementary material:**

The online version of this article (doi:10.1007/s00335-017-9723-6) contains supplementary material, which is available to authorized users.

## Introduction

Systematic high-throughput phenotyping analysis of embryonic development carried out by International Mouse Phenotyping Consortium (IMPC) revealed that 30% of the genes in mouse genome are essential for embryonic development while another 13% are considered sub-viable, because only half of the expected homozygous mutant animals are retrieved at weaning (Adams et al. 2013; White et al. 2013; Dickinson et al. 2016). In depth analysis of murine essential genes set, demonstrated statistical enrichment for the human disease genes suggesting a strong link between the genetic determinants of the normal murine embryonic development and human diseases (Dickinson et al. 2016). The systematic application of phenotyping analysis coupled to the standard annotation of developmental phenotypes curated in Mouse Genome Informatics (MGI) provides wealth of scientific knowledge for both developmental biologists and clinicians. Based on these data a specific programme, called Deciphering the Mechanisms of Developmental Disorders (DMDD), aimed to link genes with the corresponding molecular phenotypes and specific human diseases has been developed (Wilson et al. 2016; Mohun et al. 2013). The described high-throughput projects are already producing the indispensable knowledge necessary for understanding the physiology of normal embryogenesis and for discovering the genetic determinants of congenital human disorders.

Studies on embryonic development are vastly facilitated by the technological advances in the field of three-dimensional (3D) imaging methods, such as high-resolution episcopic microscopy (HREM), optical projection tomography (OPT) and X-ray micro-computed tomography (microCT) (Weninger et al. 2014, 2006; Sharpe et al. 2002; Wong et al. 2013, Hsu et al. 2016). Both strengths and limitations of these techniques when used in embryogenesis studies are described elsewhere (Norris et al. 2013). Broad application of these techniques allows for exhaustive morphometric and temporal analysis of embryonic development of normal and mutant animals with the goal to understand the anatomical details underlying the processes of normal embryogenesis and embryonic malformation or lethality.

Among volumetric imaging methods, microCT has recently gained increased popularity in biomedical research because of its relatively simple laboratory setting and powerful imaging capabilities (reviewed in Schambach et al. 2010). While microCT imaging received extensive usage in biological studies of mineralized animal tissues (Neues and Epple 2008; Schambach et al. 2010), its utilization for soft tissues analysis still represent a substantial challenge because of the low sample density which precludes a good detection in the X-ray spectrum. To circumvent this inherent constrain of the technique, several treatments with contrasting agents have been proposed in order to increase X-ray visualization of soft adult and embryonic tissues (Schambach et al. 2010; Johnson et al. 2006; Metscher 2009a, b; Gregg et al. 2015; Gregg and Butcher 2016). However, contrasting agent treatments often lead to shrinkage of the soft adult and embryonic tissues. To protect the tissues from this effect and for stabilization of the fragile murine embryos, a hydrogel treatment has been introduced prior to incubation with contrasting agent (STABILITY protocol). This experimental scheme allowed for successful implementation of high-throughput microCT imaging of embryonic and postnatal development samples (Wong et al. 2013, Hsu et al. 2016). All these technological advances encouraged the adoption of microCT imaging as a convenient and cost-effective laboratory methodology to study embryogenesis in model organisms.

Studies on earlier embryonic lethality at the stage of immediate to early post-implantation period in mice (from the embryonic day 5.5 to embryonic day 9.5) still represent a substantial challenge for developmental biologists. The main obstacle is the extreme fragility of earliest embryos which make them very hard to handle. Also, due to tight interconnection of early embryos with the maternal extra-embryonic tissues, high-quality technical skills and expertise are required for intact embryo dissection. Recently, the volumetric imaging analysis of growing murine embryos prepared at the immediate post-implantation stage (E5.5–E6.5) using light-sheet microscopy has been described (Ichikawa et al. 2014; Udan et al. 2014). However, because of the complexity of the 3D microscopy applications, the histological analysis of individual embryos still remains the most popular way to study the immediate and early post-implantation embryonic developmental stages.

In this work, we applied volume microCT imaging to study the murine embryo development at the immediate and early post-implantation period: starting from the stage of egg cylinder and the primitive streak (E5.5–E6.5), following by period of gastrulation and transformation to presomite stage embryo (E7.0–E8.5) and then from embryo turning (E8.5–E9.5) to mid-gestation (E9.5–E12.5). In order to accomplish such analysis, we performed imaging of an entire litter of developing embryos within the uterus, thus avoiding embryo dissection from the uterine extra-embryonic structures. To examine whether earliest embryos samples can provide enough contrast power for high-quality and high-resolution microCT imaging, we tested two different treatments. The first is a paraffin embedding protocol, without introduction of an additional contrasting agent while the latter is a classical treatment with potassium iodine contrasting agent (Lugol staining). Here, we show that both protocols can be applied for microCT imaging of the earliest stages of embryonic development within an entire murine conceptus. The paraffin embedding method provides sufficient endogenous contrast for high-resolution microCT imaging of both embryonic and extra-embryonic structures at very early stages of the embryogenesis, while for the mid-gestational developmental window (E9.5–E12.5) the whole-uterus potassium iodine staining protocol provides the best volumetric microCT imaging results. The volume imaging of the entire litter within the uterus would provide embryologists with a tool for a thorough characterization of embryonic development that takes in account the spatial and morphological interactions between maternal and foetal tissues. Indeed, from such analysis is achievable a one shot evaluation of several developmental parameters like the foetal orientation within the uterus, the anterior/posterior and dorsal/ventral axis formation, the allantois and placental development among others.

To study earliest embryonic lethality in murine mutant knockout lines, we developed a two-step phenotyping scheme. First, the interval in which the development has been arrested is evaluated by a low-resolution microCT imaging analysis of the entire litter (19 µm/voxel). In the second step, embryos with evident developmental delays are examined with high-spatial resolution of 1.4 µm/voxel to reveal and characterize specific developmental defects. To validate this approach, we analyzed the mouse line with the knockout of tRNA endonuclease subunit Tsen54. *Tsen54* gene is essential for early embryonic development since we did not retrieve any Tsen54 null animals at birth. MicroCT imaging of entire litters at different developmental points revealed that Tsen54 null embryos were able to implant into uterine wall, but did not develop beyond implantation. Our results suggest that the proposed whole-uterus microCT imaging approach will greatly facilitate studies aimed to determine the precise period in which early embryonic lethality occurs during embryogenesis and can be implemented into high-throughput phenotyping efforts aimed to understand the mechanisms by which mammalian genes drive the embryogenesis processes.

## Results

### MicroCT analysis of the murine immediate post-implantation development within the uterine tissues in C57BL6/N mouse strain

**Fig. 1:**
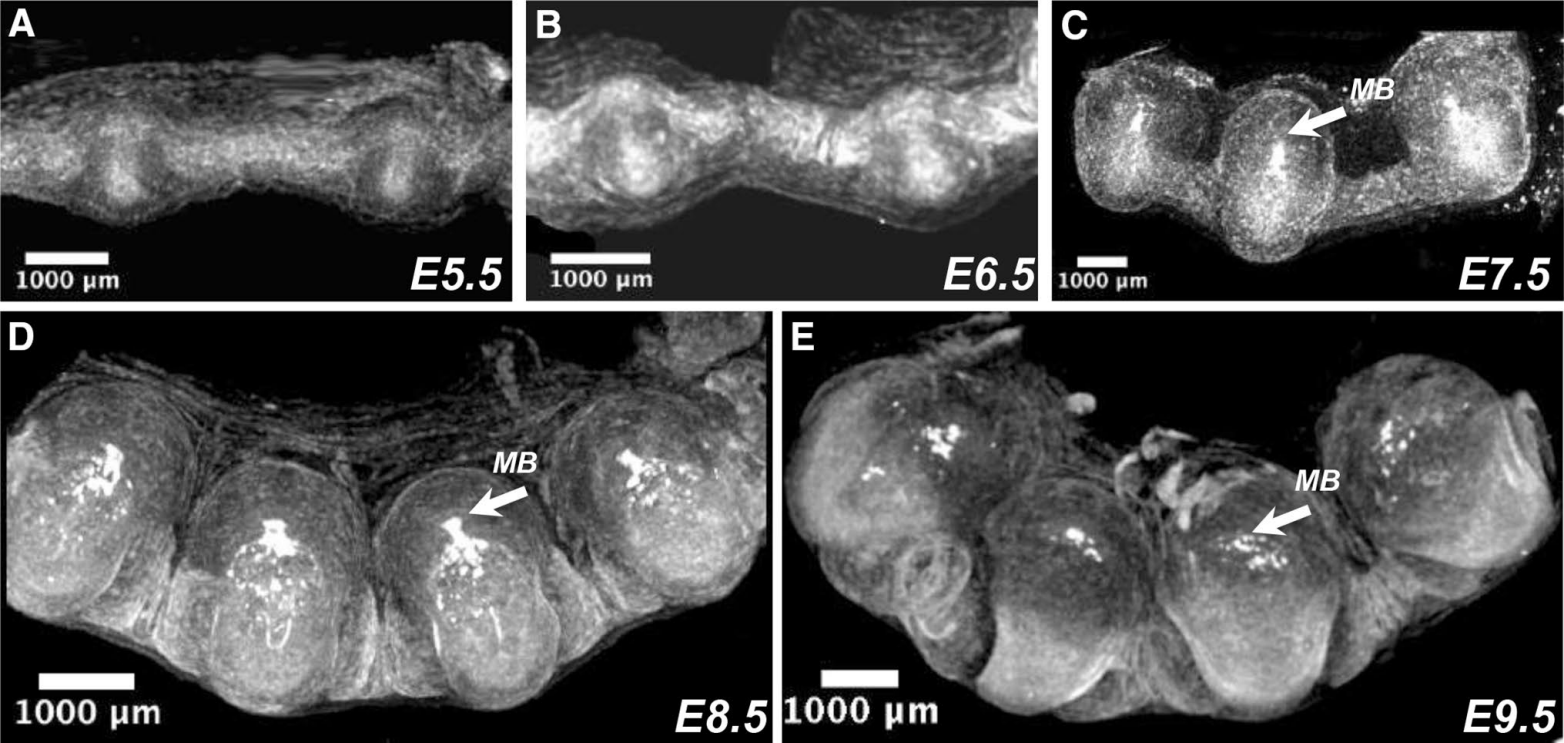
Maximum intensity projection (MIP) images of murine gravid uteri at different days after fertilization (p.c.) imaged with the 19 µm/voxel resolution. **a** Embryonic day 5.5, (E5.5); **b** embryonic day 6.5 (E6.5); **c** embryonic day 7.5 (E7.5); **d** embryonic day 8.5 (E8.5); **e** embryonic day 9.5 (E9.5). Arrows indicate maternal blood cells (MB) within developing decidua

To demonstrate that the microCT imaging could be applied to study earliest stages of mouse embryonic development such as immediate post-implantation, gastrulation and early organogenesis without preparative dissection of individual embryos from the maternal structures, we performed microCT imaging of entire litters within the uterine tissues of C57BL/6N mouse strain. The gravid uteri between E5.5–E9.5 were embedded in paraffin and then imaged by microCT. We found that the uterine walls protect both embryonic and extra-embryonic maternal tissues surrounding the developing embryos from being damaged during sample preparation. Moreover, the paraffin moiety provides a rigid support so facilitating sample mounting for the microCT imaging. The paraffin embedded uteri were imaged first at low-resolution of 19 μm/voxel (Fig. 1). We also tried to image E4.5 pregnancy containing uteri at 19 µm/voxel resolution. While we observed a uterine lumen closure indicative of a fertilization occurrence, we were not able to detect the individual attachment sites at this resolution (data not shown). In addition, we demonstrated that the whole-mount uterus paraffin embedding could not be applied for the stages of development after E9.5 because of extra-embryonic membranes rupture and uterine walls collapse caused by this treatment. Overall, we performed whole-uterus scanning from the E5.5–E9.5 in C57BL6/N strain of 110 embryos (two pregnancies for every day of post conception) and observed that in wild type animals 3.6% of deciduas resulted empty or contained embryos visibly delayed in development or in the process of resorption. Our results are in agreement with previously published studies indicating a 4% of spontaneous resorption when gastrulation period were assayed in random breed albino mice (Downs and Davies 1993).

### Volume imaging of the egg cylinder stage of embryonic development

**Fig. 2:**
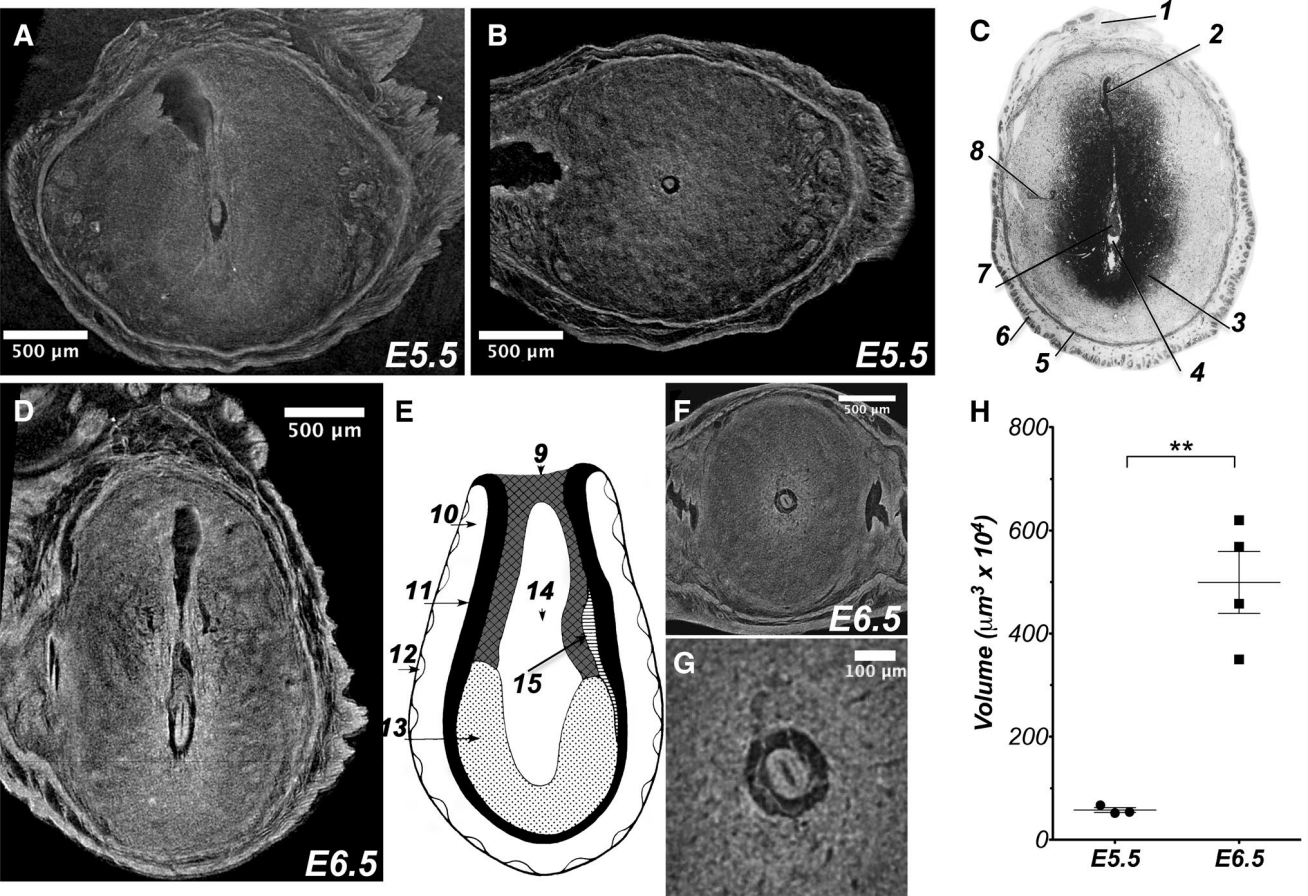
3D microCT analysis of murine immediate post-implantation development within conceptus at E5.5 and E6.5 days. **a, b** High-resolution (1.4 µm/voxel) 2D virtual sections of volume reconstructed microCT images of E5.5 murine conceptus with the embryo at egg cylinder stage, Theiler Stage (TS08); sagittal (**a**) and transverse (**b**) sections. **c** Histological analysis of E5.5 conceptus from the Atlas of Mouse Development (Kaufman 1999) page 24, panel **a**; *1* mesometrium; *2* remnant of uterine lumen; *3* zone of decidual reaction; *4* yolk sac cavity; *5* inner circular layer of myometrial smooth muscle; *6* outer longitudinal layer of myometrial smooth muscle; *7* egg cylinder stage embryo at centre of decidual reaction; *8* uterine glands. **d** High-resolution (1.4 µm/voxel) 2D virtual sagittal section of volume reconstructed microCT images of E6.5 conceptus with the embryo at advanced egg cylinder stage (TS09). **e** Schematic representation of the advanced egg cylinder stage embryo from The Atlas of Mouse Development (Kaufman 1999) page 12; Fig. 1a: *9* ectoplacental cone; *10* yolk sac cavity; *11* visceral endoderm; *12* parietal endoderm; *13* embryonic endoderm; *14* amniotic cavity; *15* intra-embryonic mesoderm. **f** Transverse 2D virtual section of the 3D reconstructed E6.5 murine conceptus. **g** Magnified transverse section from panel **f. h** Quantification of the volume occupied by the embryo within conceptus at E5.5 and E6.5 developmental days; Student’s *t* test **P < 0.005

In order to demonstrate the potential of microCT imaging for detailed analysis of immediate post-implantation development and to obtain high-resolution volume images, we scanned conceptuses with the resolution as high as 1.4 μm/voxel. We first focused on the analysis of egg cylinder stages of murine embryonic development (E5.5 and E6.5) (Fig. 2). Volume images of paraffin embedded pregnancies demonstrated that embryos at egg cylinder stage can be visualized at high-resolution using endogenous tissue contrast. Virtual microCT derived two-dimensional (2D) sections from these experiments can be compared and aligned to the histological sections of The Atlas of Mouse Development (Kaufman 1999). The Atlas of Mouse Development is the main reference for developmental biologists studying murine embryonic development and is also adopted in our studies. The digital 2D sections obtained from full 3D reconstructed volumes demonstrated that the main structures of the developing egg cylinder embryo, such as developing proamniotic cavity and yolk sac cavity surrounding the embryo, are well preserved and in striking agreement with the corresponding histological sections of the Atlas of Mouse Development (Fig. 2a–c). The uterine smooth muscles, uterine glands and decidua were shown in microCT images in great detail allowing for accurate analysis of parental-embryonic interactions and orientation of the embryo within the developing decidua and uterus (Supplementary Movie 1).

Based on the Atlas of Mouse Development, embryos at the embryonic day 6.5 are staged as an advanced egg cylinder (Fig. 2d–g). The main features that characterize the transformation from egg cylinder stage to the advanced stage is the growth of the ectoplacental cone and the proamniotic cavity and well formed ectoderm and endoderm layers, all these features can be visualized by microCT imaging (Fig. 2d–g and Supplementary Movie 2).

Our experimental protocol coupled to microCT imaging allows for an in depth morphometric analysis of developing embryos. To illustrate this, we measured the total volume occupied by the developing embryos at E5.5 and E6.5 and demonstrated that about a 12 fold increase of the total embryonic volume occurs in a single day at this stage of development (Fig. 2h). This is in good agreement with the previously published data (Rands 1986).

### MicroCT imaging of murine gastrulation

**Fig. 3:**
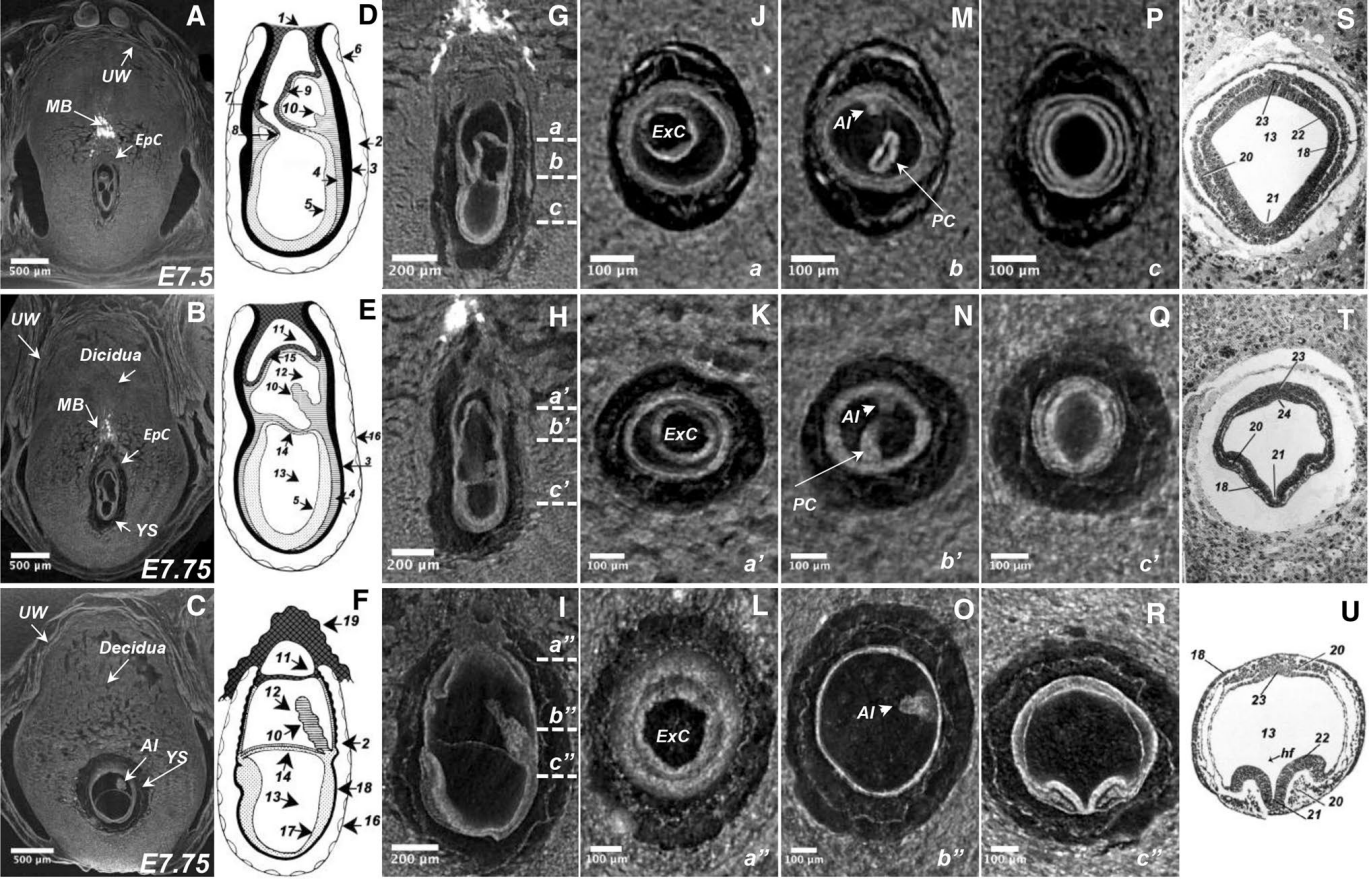
Three-dimensional microCT imaging of murine gastrulation period. **a**–**c** Virtual 2D section derived from reconstructed volume of murine conceptuses imaged during gastrulation period (frontal view) with the resolution of 1.4 µm/voxel: E7.5, advanced TS10 (**a**) and E7.75 staged as TS11 (**b**–**c**). **d**–**f** Schematic representation of the embryonic development during stages of gastrulation from The Atlas of Mouse Development (Kaufman 1999), page 12, Fig. 1: proamniotic canal present (**d**); proamniotic canal closed (**e**) and presomite head fold embryo (**f**). **g**–**i** Sagittal 2D virtual sections of the embryos at different stages of gastrulation in correspondence to the schematic representation on the left; *a, b, c; a’, b’, c’* and *a”, b”, c”* indicate the planes of the transverse sections. **j**–**r**. Transverse 2D virtual sections derived from the volume images of murine conceptuses (respective planes of the sectioning are indicated by small letters at the low-right corner). **s**–**u** Histological sections of the mouse embryo from The Atlas of Mouse Development aligned to 2D microCT image from adjacent left panel of the figure. **s** The Atlas of Mouse Development page 32 panel **i; t** page 34 panel **n; r** page 36 panel **f**. *1* ectoplacental cone; *2* yolk sac cavity; *3* visceral endoderm; *4* mesoderm; *5* embryonic ectoderm; *6* parietal endoderm; *7* proamniotic canal; *8* anterior amniotic fold; *9* posterior amniotic fold; *10* allantois; *11* ectoplacental cavity; *12* exocoelomic cavity; *13* amniotic cavity; *14* amnion; *15* chorion; *16* reichert’s membrane; *17* embryonic ectoderm and mesoderm; *18* embryonic endoderm; *19* ectoplacental cone; *20* intra-embryonic mesoderm; *21* neural groove; *22* neural ectoderm; *23* caudal region of notochordal plate; *24* primitive groove; *hf* head fold; *MB* maternal blood; *YS* yolk sac; *EpC* ectoplacental cone; *Al* allantois; *UW* uterine wall; *ExC* exocoelomic cavity; *PC* proamniotic canal

To study period of gastrulation we imaged the conceptuses at embryonic day 7.5 and 7.75. Embryos at E7.5 were collected in the morning (around 9 a.m.) of day 7 after p.c. (post-coitus) as described in Materials and Methods, while the E7.75 embryos collection was performed about 4 p.m. of day 7 after p.c. MicroCT imaging of the paraffin embedded conceptuses were performed at a resolution of 1.4 µm/voxel (Fig. 3 and Supplementary Movies 3–5).

During gastrulation, the embryonic development is characterized by conversion of primitive streak embryo to the head fold embryo (Fig. 3a–c). The critical steps of gastrulation are schematically presented in Fig. 3d–f (from The Atlas of Mouse Development). This process begins with the expansion of both posterior and anterior embryonic folds (Fig. 3d, g) followed by their fusion, which produces amnion and chorion and divides unique proamniotic cavity into three separated cavities: ectoplacental, exocoelomic and amniotic (Fig. 3e, h and f, i). Transverse digital 2D microCT sections through the extra-embryonic portion of the developing embryo demonstrate formation of exocoelomic cavity during gastrulation process (Fig. 3j–l). The transverse sections performed at the junction between embryonic and extra-embryonic parts of the developing embryo, show the process of the proamniotic canal closing, formation and expansion of allantois (Fig. 3m–o). Such in Fig. 3m, proamniotic canal is open and allantoic bud is very small, while in Fig. 3n, the amniotic canal is in the closing step and allantois is visibly increased in size. Figure 3b, h demonstrate that the formation of amniotic, exocoelomic and ectoplacental cavities are at completion, and in Fig. 3o, the exocoelomic cavity with a well developed allantois protruding into the cavity is clearly visible. To illustrate the formation of the head fold, we compared transverse microCT digital 2D sections obtained from the embryonic portion of the developing embryo (Fig. 3p–r) to the best corresponding histological sections of The Atlas of Mouse Development (Fig. 3s–u). The three embryonic layers: ectoderm, mesoderm, and endoderm as well as neural groove and forming head fold could be recognized in transverse microCT sections taken from embryos at gastrulation stage (Fig. 3p compared to Fig. 3s, Fig. 3q compared to Fig. 3t); formation of the head fold became completed at the E7.75 (Fig. 3r compared to Fig. 3u).

We also tested whether potassium iodine (Lugol) staining, when applied to the gravid uteri at the earliest stages of embryonic development, can provide a sufficient contrast for morphometric analysis by microCT. We observed that the uterine walls did not preclude the stain penetration into the embryonic and decidual tissues and that, importantly, both embryonic and extra-embryonic structures acquired sufficient contrast upon potassium iodine treatment to be visualized by microCT imaging (Supplementary Fig. 1). We, however, also observed that the paraffin embedding provides a better contrast for structures derived from both embryonic and extra-embryonic components of the developing embryos at earliest stages of murine embryonic development making the whole-uterus paraffin embedding preparation for microCT imaging the methods of choice to achieve high-resolution, high-quality imaging of earliest stages of murine embryonic development.

### Three-dimensional whole-uterus imaging from post-gastrulation to organogenesis (E8.5–E12.5) period

**Fig. 4:**
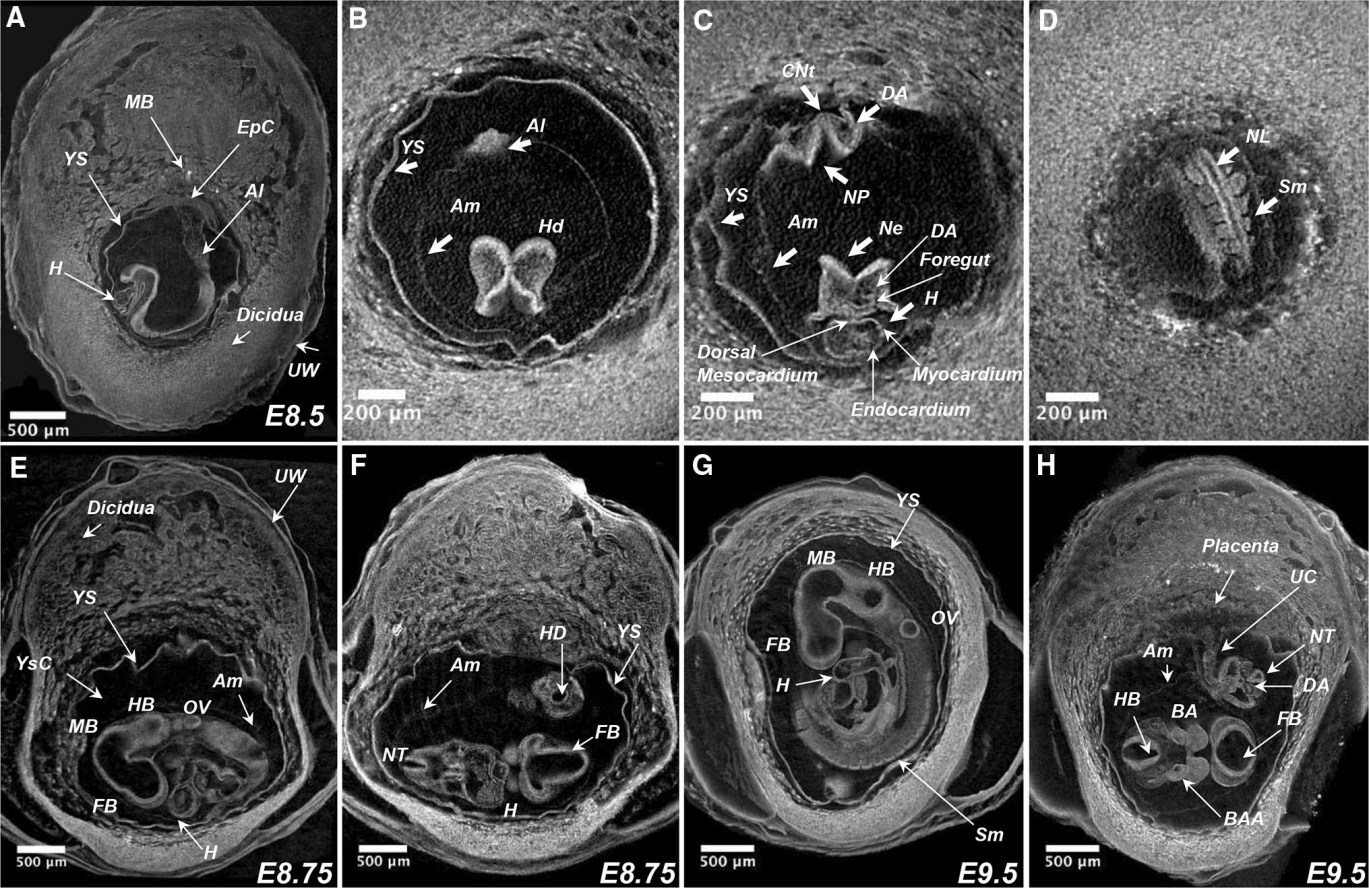
3D imaging of embryonic post-implantation development. **a**–**c** 2D sections of the microCT produced volume images of the murine conceptus at E8.5 (TS12; 7 pairs of somites) with resolution of 3.9 µm/voxel: sagittal section (**a**); transverse view of the primitive head region (**b**); transverse view through the forming heart plane (**c**); transverse view of the somites and neural lumen (**d**). **e, f** 2D virtual sections of the murine conceptus at E8.75 (TS14; 14 pairs of somites) imaged at resolution 3.9 µm/voxel; sagittal (**e**) and coronal (**f**) views. **g, h** Virtual 2D sections through the volume image of the murine conceptus at E9.5 (TS15; 23 pairs of somites) imaged at 3.9 µm/voxel resolution; sagittal (**g**) and coronal (**h**) views. *YS* yolk sac; *YsC* yolk sac cavity; *Al* allantois; *UW* uterine wall; *EpC* ectoplacental cone; *Am* amnion; *Hd* head; *CNt* caudal notochord; *DA* dorsal aorta; *NP* neural plate; *Ne* neuroepithelium in prospective hindbrain; *H* heart; *NL* neural lumen; *Sm* somites; *OV* otic vesicle; *HB* hindbrain; *MB* midbrain; *FB* forebrain; *NT* neural tube; *HD* hindgut diverticulum; *BA* first branchial arch; *BAA* first branchial arch artery; *UC* umbilical cord

Following the developmental analysis, we imaged the whole-mount paraffin embedded conceptuses first at embryonic day 8.5 at 1.4 µm/voxel resolution (Fig. 4a–d). The high endogenous contrast derived from the tissues allowed us to visualize both embryonic and extra-embryonic structures at this stage as well. The virtual 2D sections through the volume reconstructed illustrate the main developmental features of the 8.5 days embryo (Fig. 4a–d). At this stage, the allantois, heart and the somites are well defined (Fig. 4a–c). Dorsal aorta and foregut are clearly visible and in the forming heart final anatomical features such as dorsal mesocardium by which the heart tube is attached to the body, myocardium and endocardium can be distinguished (Fig. 4c). The sharp endogenous differential tissue contrast allowed for somite count and embryo staging. As an example, Fig. 4d shows an embryo with 7 pairs of somites.

At embryonic day 8.75 (embryos were collected at 4 p.m. at day 8 of p.c.), embryo has 14 pairs of somites, the process of turning is almost completed and the extra-embryonic structures progressed further through development; at this stage, very well developed placenta and umbilical cord are formed (Fig. 4e, f). There are no dramatic developmental changes observed when embryo E8.75 and E9.5 embryos are compared as a gradual development of the all anatomical structures proceeds (Fig. 4g, h). At E9.5, we counted 23 pairs of somites (Fig. 4g).

**Fig. 5:**
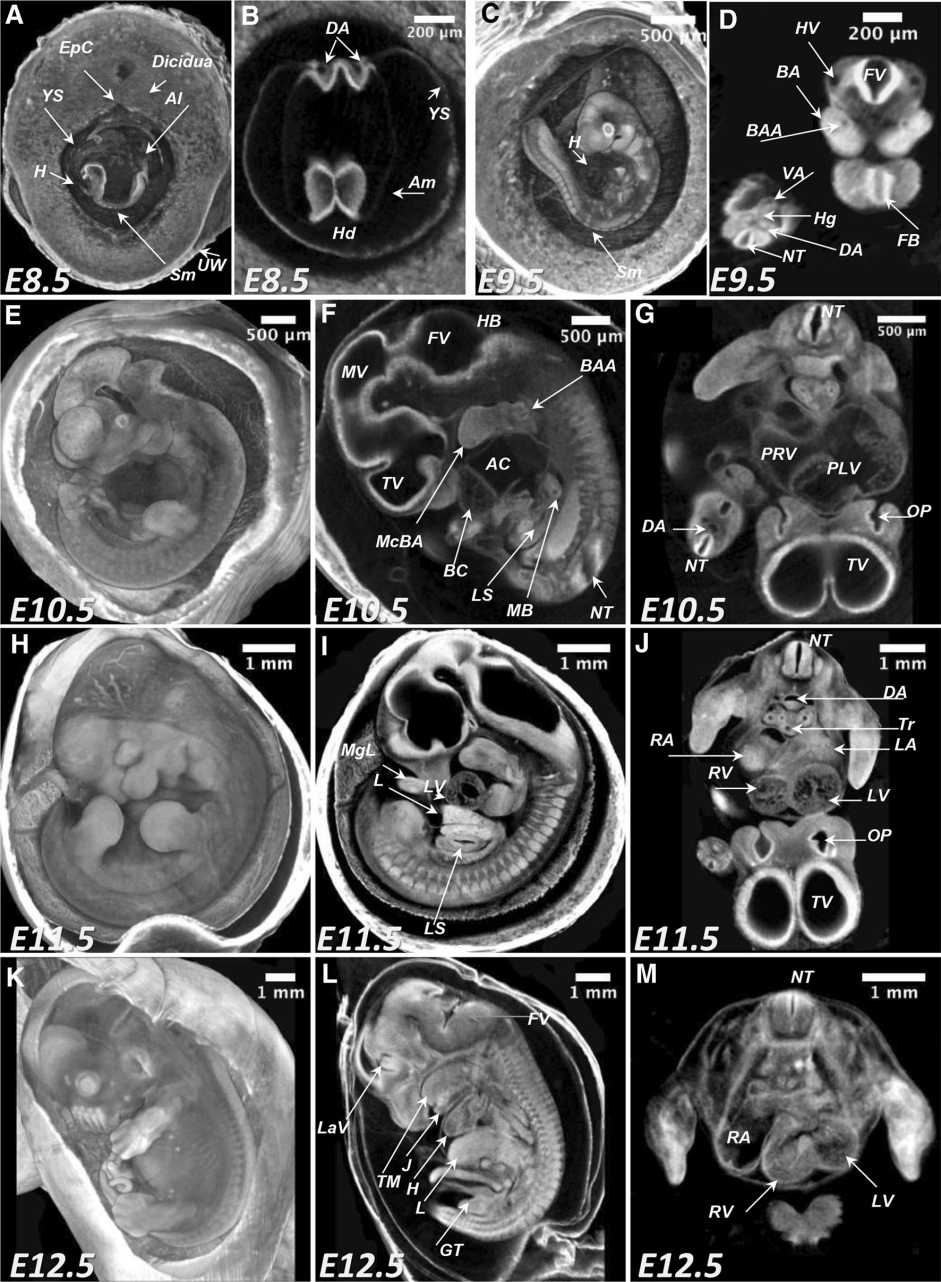
Whole-uterus microCT imaging of the murine conceptuses from E8.5–E12.5 treated with potassium iodine contrast. **a, b** MicroCT derived volume image of the E8.5 (TS13; 8 pairs of somites) at 3.9 µm/voxel resolution. Virtual 3D volume rendering (**a**) and transverse section at the level of the forming embryonic head (**b**). **c, d** MicroCT derived volume of the E9.5 (TS15; 21 pairs of somites) conceptus imaged at 3.9 µm/voxel. Virtual 3D volume rendering (**c**) and transverse (**d**). **e**–**g** MicroCT derived volume image of the E10.5 (TS16-17) conceptus at 3.9 µm/voxel resolution: 3D volume rendering (**e**), sagittal (**f**) and transverse (**g**) 2D virtual sections. **h**–**j** Digital images of the E11.5 (TS18) conceptus imaged with the 4.4 µm/voxel resolution: 3D volume rendering (**h**); sagittal (**i**) and transverse (**j**) 2D virtual sections. **k**–**m** Digital images of the E12.5 (TS20) conceptus imaged with the 7.9 µm/voxel resolution: 3D volumes rendering (**k**); sagittal (**l**) and transverse (**m**) 2D virtual cross sections. *YS* yolk sac; *H* heart; *Al* allantois; *UW* uterine wall; *EpC* ectoplacental cone; *Sm* somites; *Hd* head; *DA* dorsal aorta; *Am* amnios; *Hg* hindgut; *VA* vitelline artery; *BA* branchial arch; *BAA* branchial arch artery; *HV* head vein; *FV* fourth ventricle; *NT* neuronal tube; *FB* forebrain vesicle; *TV* telencephalic vesicle; *MV* mesencephalic vesicle; *HB* hindbrain (roof); *AC* atrial chamber of heart; *McBA* mandibular component of first branchial arch; *BC* bulbus cordis; *MgL* midgut loop with the umbilical hernia; *LS* lumen of stomach; *MB* main bronchus; *PRV* primitive right ventricle; *PLV* primitive left ventricle; *OP* Olfactory pit; *L* liver; *RA* right atrium; *LA* left atrium; *RV* right ventricle; *LV* left ventricle; *LaV* lateral ventrical; *Tr* trachea; *TM* tongue muscles; *J* jaw (lower); *GT* genital tubercle

Overall, the whole-uterus paraffin embedding protocol provides an excellent contrast allowing for the assessment of fine structures inside developing organs such as dorsal aorta, neuronal tube, branchial arches, developing brain and heart ventricles as well as extra-embryonic structures such as amnios, yolk sac, umbilical cord and placenta (Fig. 4). However, as we stated above, this protocol cannot be applied after embryonic day 9.5 because of the extra-embryonic structures collapse during the embedding procedure. Therefore, we tested whether the application of the potassium iodine contrast for imaging of the whole conceptus could be a good alternative to paraffin embedding. Whole litter staining with the potassium iodine was performed from E8.5 till embryonic day E12.5 (Fig. 5 and Supplementary Movies 6–10). Comparison of the images obtained from paraffin embedded and potassium iodine stained embryos for the E8.5 and E9.5 demonstrated that application of both protocols provide high-quality images with excellent contrast (Figs. 4, 5a–d).

Further analysis of murine conceptuses at later stages of development showed that the organogenesis can be analyzed in details when iodine contrast (Lugol solution) is applied for the mid-gestational developmental period (E8.5–E12.5) (Fig. 5). Differential contrast derived from the developing tissues after Lugol staining allows for the morphometric analysis of organogenesis such as developing heart, brain, liver, stomach and skeletal apparatus.

**Fig. 6:**
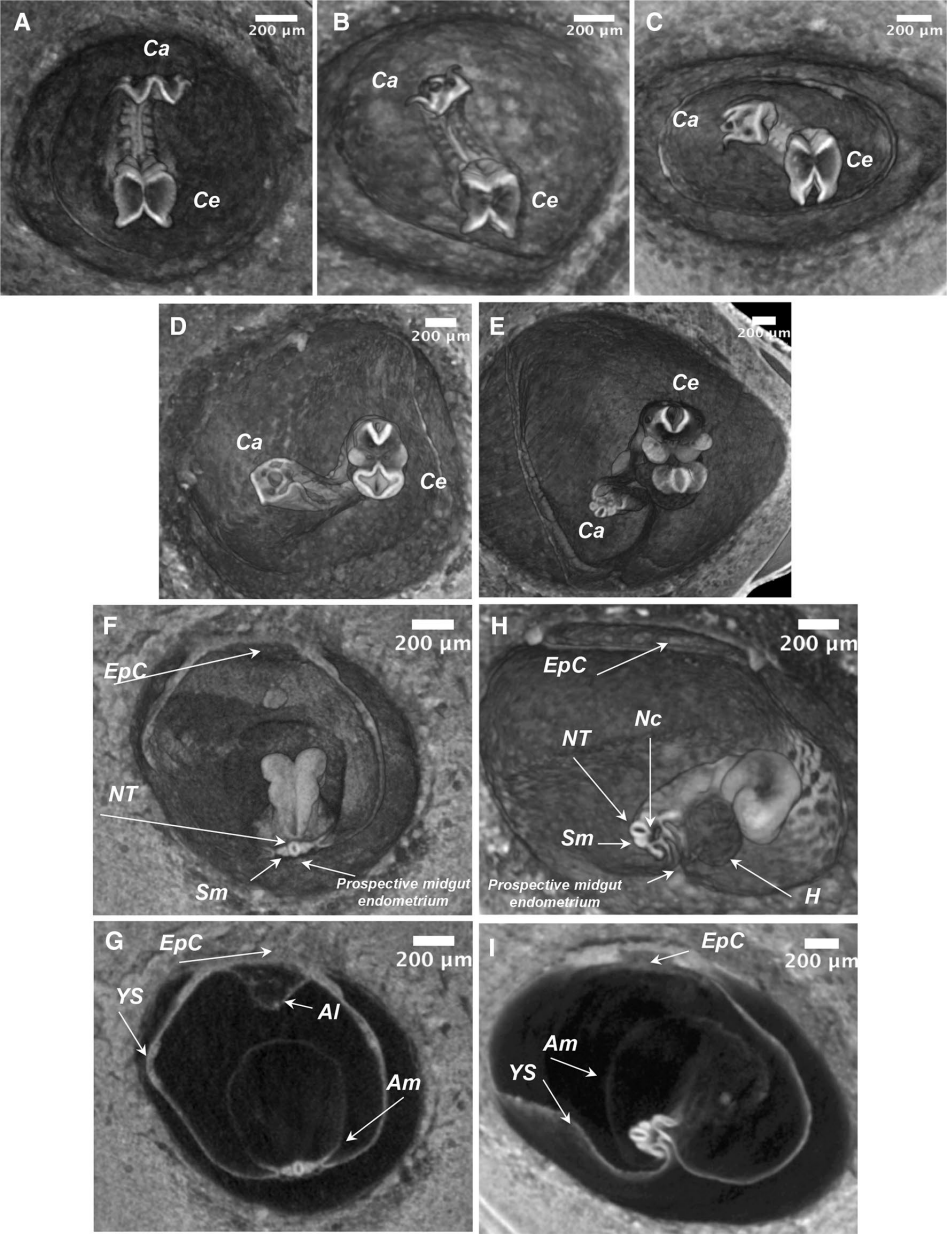
Three-dimensional microCT imaging of embryo “turning” process in C57Bl6/N animals. **a**–**e** Transverse sections from the 3D volumes through the caudal and cephalic planes of embryos undergoing “turning” (3.9 µm/voxel resolution). “Unturned” embryo at E8.5 of development with 8 pairs of somites (**a**). Embryo initiated the rotation along the body axis; embryo with 10 pairs of somites (**b**); rotation proceed anticlockwise at this stage the embryo is rotated about 90°; embryo with 11 pairs of somites (**c**); and more than 100° embryo with 12 pairs of somites (**d**). The process of “turning” is almost completed and rotation close to 180° is observed; This embryo at E9.5 with 24 pairs of somites (**e**). **f**–**i** Coronal sections through the mid-trunk region of the volume reconstructed conceptuses presented to visualize positions of extra-embryonic membranes during turning. **f, g** Unturned embryo, the same as in panel **a**: volume rendering (**f**) and 2D view of the volume section (**g**). **h, i** Embryo is advanced in “turning”; the stage presented in panel **d**: volume rendering (**h**) and 2D view of the volume section (**i**). *Ca* caudal part of embryo; *Ce* cephalic part of embryo; *EpC* ectoplacental cone; *NT* neural tube; *Nc* notochord; *Sm* somites; *Al* allantois; *Am* amnion; *YS* yolk sac; *H* heart

The embryo “turning” process or axial rotation is a process which starts when embryo has about 6–8 pairs of somites. As a result of this process, the mouse adopts, common for all other chordates, a foetal position. Here, we applied volume microCT imaging to visualize murine embryo “turning” sequence. In the U-shaped “unturned” embryo stage, its dorsal surface is located within the “U” and the cephalic and caudal parts of the embryo are opposing each other at the extremities of the “U” (Kaufman 1999). The sequence of events that illustrates embryo rotation of 180° anticlockwise imaged by microCT is presented on Fig. 6a–e. These images, obtained from different embryos, show specific steps in turning process. Upon the completion of the “turning” sequence, the dorsal surface (neural tube) of the embryo turns to the outer part of U, while the midgut region moves insight (compare Fig. 6f–h). The other landmark of the axis rotation is that the embryo becomes surrounded by its extra-embryonic membranes as shown in Fig. 6g, i. Therefore, here, we illustrated how volume microCT imaging can be instrumental for studies on the embryo turning process in normal and mutant mice.

Thus, our results demonstrate that the 3D microCT analysis is an excellent tool for systematic studies of the murine early embryogenesis course and its systematic application will provide an opportunity to generate a virtual anatomical atlas of the murine early embryogenesis for a fast and quantitative evaluation of the morphological features changes during development, a useful complement of the studies already performed on the later stages of embryonic growth (Wong et al. 2012, 2015).

### Application of microCT imaging to study embryonic lethality during immediate and early post-implantation period

**Fig. 7:**
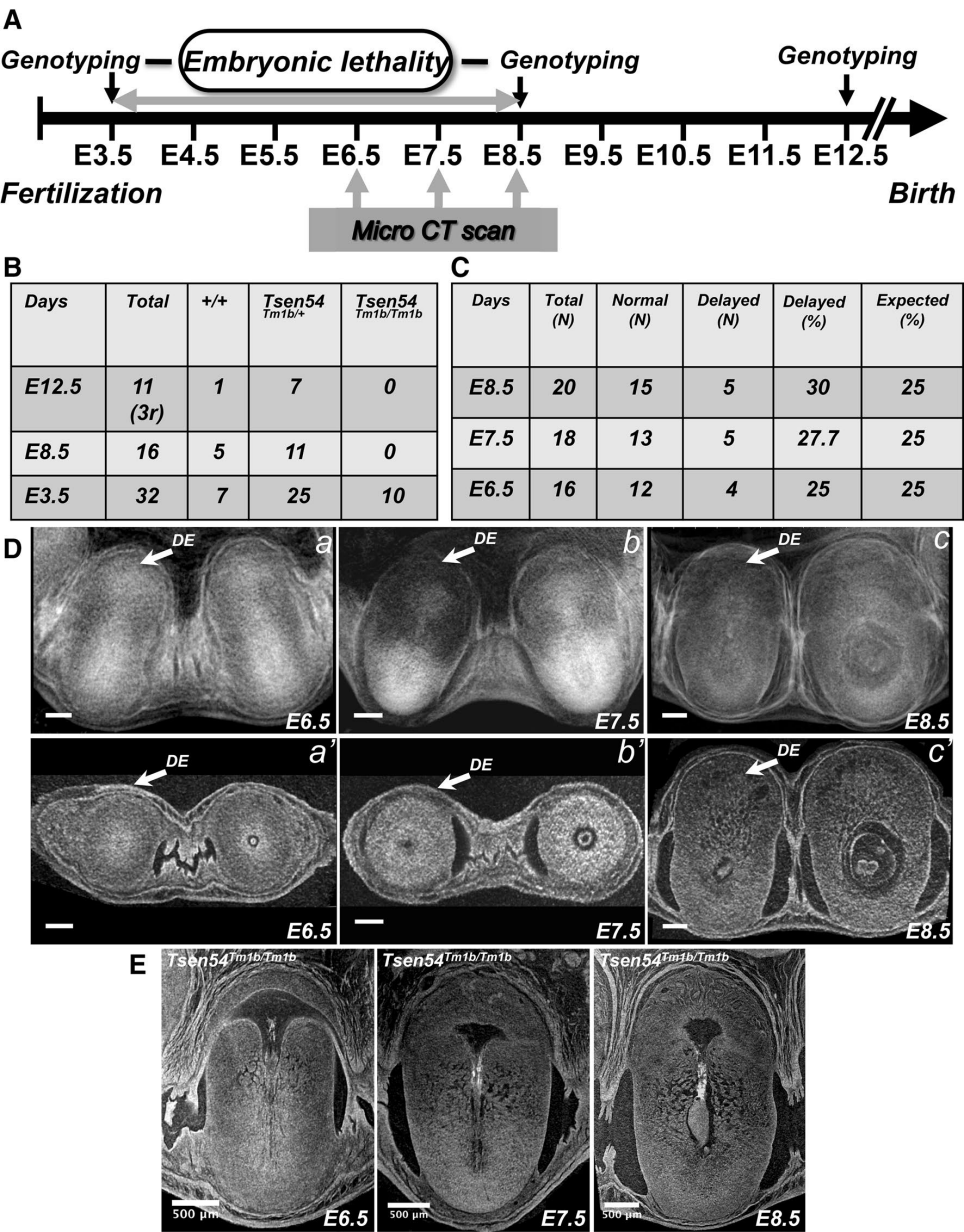
Phenotyping analysis of embryonic lethality in Tsen54 null animals. **a** Schematic representation of phenotyping analysis performed to determine the interval of early embryonic lethality. The developmental window in which the embryonic lethality occurs is first determined by genotyping. The microCT imaging of the gravid uteri is performed to determined developmental window at which delayed embryos can be visualized. **b** Summary of the genotyping analysis performed on the littermates obtained from *Tsen54* ^*tm1b/+*^ heterozygous breeding (r in process of resorption). **c** Summary of the microCT imaging analysis of the pregnancies derived from the *Tsen54* ^*tm1b/+*^ heterozygous crosses. The embryos with the developmental delay are detected in mendelian ratio as early as E6.5 of development. **d** The representative microCT scans of normal and delayed embryos with the uterine tissues at resolution of the 19 µm/voxel: (*a, b, c*) volume images at indicated interval of the embryonic development (radiogram modality); (*a’, b’, c’*) 2D virtual cross sections of volume reconstructed gravid uteri. **e** The 2D cross sections from the reconstructed high-resolution volume images (1.4 µm/voxel) of the *Tsen54* ^*tm1b/tm1b*^ null embryos at E6.5, E7.5 and E8.5 developmental days

We asked whether our whole-uterus volume microCT imaging approach could be applied to study early post-implantation lethality in the mutant animals. With this aim, we developed a two-step phenotyping scheme in order to determine the temporal window of the earliest embryonic death and to characterize morphological abnormality of both embryos and extra-embryonic structures in mutants (Fig. 7a). We applied our protocol to a mouse line with the knockout for tRNA endonuclease subunit 54 gene, *Tsen54* (*Tsen54* ^*tm1b*/*tm1b*^ mouse line; Supplementary Fig. 2). The Tsen54 protein is a subunit of the tRNA splicing endonuclease complex and is required for removal of introns from tRNA and for the pre-mRNA maturation. It has been shown to be an essential gene for viability in yeast, *Saccharomyces cerevisiae* (Dhungel and Hopper 2012). We first demonstrated that the*Tsen54* gene is essential for the murine embryonic development. The breeding *Tsen54* ^tm1b/+^ heterozygous animals did not produce any viable *Tsen54* null pups after birth. In order to determine the developmental window in which the embryonic lethality occurs, we performed genotyping analysis of the embryos at different points of embryonic development (Fig. 7b). Genotyping of the embryos at E3.5 demonstrated that the *Tsen54* null blastocysts are produced in Mendelian ratio (Fig. 7b). However, we did not recover viable *Tsen54* null embryos at E8.5 suggesting that the embryonic death occurs between E3.5 (blastocyst stage) and E8.5 of development. At this particular developmental interval, it is very challenging to isolate embryos from the maternal tissues for morphological analysis and genotyping. This is due to small size of the embryos at this stage, especially when mutant embryos show a delay in development or resorption already initiated. In this case, the genotyping of individual mutant embryos become impossible because they are contaminated by invading maternal cells.

To determine with the greater precision, the developmental point at which embryogenesis is arrested, we performed first at low-resolution (19 µm/voxel), ex vivo whole-uterus microCT scanning of the murine pregnancies in the determined developmental interval. Even with this low-resolution of microCT analysis, we were able to recognize defective embryos and calculate if the number of delayed embryos presented in expected for homozygous null allele segregation Mendelian ratio (Fig. 7c, d). Starting from the day 6.5 till the day 8.5, we observed about 25% percent of abnormal/delayed embryos, suggesting that the development of the homozygous embryos is arrested at immediate post-implantation period.

After, we performed high-resolution microCT analysis (1.4 µm/voxel) focusing only on abnormal conceptuses selected at low-resolution and demonstrated that already at E6.5 of development, null embryos failed to form properly. Embryonic structures that are clearly built in normally developing littermates and in wild type embryos (Fig. 2d–e) are absent in the arrested *Tsen54* null embryos (Fig. 7e). However, the extra-embryonic tissues are formed normally and are undistinguishable from the wild type embryos (Fig. 2d–e). Indeed, it has been previously shown that even highly compromised embryos can trigger decidualization of the uterine tissues as long as they shed the zona pellucida and had a differentiated trophoblast layer (Papaioannou and Behringer 2012). The growth of decidual tissues in *Tsen54* null embryos proceeds without major morphological defects up to embryonic day E8.5 (Fig. 7e) even in the absence of developing embryos. We also observed an increase in unstructured tissues within the embryonic cavity at E8.5, suggesting that the null embryo resorption was in progress. These results are in agreement with the observation that no homozygous *Tsen54* null embryos were recovered by genotyping analysis at E8.5 (Fig. 7b).

We concluded that the development of *Tsen54* null animals is arrested between E4.5–E5.5, at peri-implantation period (Fig. 7). This result suggests that our phenotyping scheme, which adopts whole-uterus microCT imaging of the littermates, can be instrumental to determine the temporal interval of the earliest embryonic lethality in mutant animals and to conveniently replace labour consuming histological analysis currently in use in most laboratories.

## Discussion

In this work, we showed how X-ray imaging of unperturbed murine conceptuses by a laboratory microCT system can be implemented to study the normal embryonic development as well as for phenotyping analysis of mutant animals from immediate post-implantation to mid-gestation period (E5.5–E12.5). The main advantage of the proposed method is that the embryos are imaged within the maternal uterine tissues, allowing for a simultaneous analysis of embryonic and maternal components and their relationship during embryogenesis. The sample preparation for volume imaging presented here does not require dissection of the individual embryos from the maternal uterus, which greatly facilitate the analysis of embryogenesis. This is especially critical for studies of the earliest stages of development when dissection of the individual embryos is very tedious and requiring optimal handling skills. Since microCT imaging is non-invasive, multiple scanning can be performed without destructive consequences for the samples. Therefore, to make the analysis more efficient, we introduced a two-step scheme. At first, a low-resolution scan allows, in approximately one hour microCT scan, to recognize samples with distinguishable abnormalities within conceptus. A detailed morphometric analysis can be later performed on individual chosen embryos, at higher spatial resolution (up to 1.4 µm/voxel for the E5.5–E9.5 and about 3.9–7.9 µm/voxel resolution for the stages from the E10.5–E12.5) requiring about only 2 h for every embryo examined. While the commercially available laboratory microCT systems do not allow for cellular resolution analysis, here we demonstrated that the main structural features of early embryogenesis can be easily identified and morphometric analyses of the embryonic and extra-embryonic structures can be performed with good accuracy. 3D volume microCT images can be digitally 2D sectioned and checked for correspondence with related histological sections of The Atlas of Mouse Development. Such comparison allows for a faithful characterization of anatomic structures of the developing embryos, providing an excellent alternative to the classical time-consuming histological analysis. The presented experimental scheme greatly facilitated the analysis in early embryogenesis studies aimed to uncover the genetic and environmental determinants of the normal embryonic development and its perturbations.

In this work, we have tested two protocols for the whole-conceptus preparation for microCT imaging of the early embryogenesis from E5.5–E12.5: paraffin embedding and potassium iodine contrasting agent (Lugol) staining protocol. The whole-uterus paraffin embedding is the method of choice to perform morphometric analysis of the early embryogenesis from E5.5 to E9.5. This method provides excellent non-destructive way to obtain the high-quality and high-resolution microCT imaging without the need for the contrast agents. Even for the earliest conspectuses analyzed at immediate post-implantation stage, the embedded paraffin provides sufficient differential density contrast to allow visualization of the developing embryonic structures and tissue layers. The small embryo structures and extra-embryonic tissues are very stable when are embedded in paraffin, can be scanned multiple times and stored for a long time (even for more than 1 year in our experience). It is worth to note that in addition to the simplicity of the protocol, the whole-uterus paraffin embedded embryos are ready for an eventual follow-up analysis such as the traditional histological analysis, in situ hybridization and immunofluorescence analysis. Recently, several protocols were developed and kits are commercially available for DNA and RNA extraction from the formaldehyde fixed and paraffin embedded tissues for further biochemical analysis (Martelotto et al. 2017; Kotorashvili et al. 2012). The combination of morphometric analysis with a post-imaging in depth biochemical analysis will provide a multilayer approach to understand the molecular mechanisms of congenital malformations and developmental diseases.

The whole-uterus wax-embedding protocol, however, cannot be applied for the an entire litter analysis after E9.5. The uterine structures break and collapse during paraffin embedding after E9.5 hampering the application of this paraffin-based protocol at later stages of development. Therefore, for the period of mid-gestation (E9.5–E12.5), we implemented the iodine staining protocol. Potassium iodine provides a X-ray contrast for the soft tissues and it is the contrasting agent of choice for the analysis of the embryonic development in mice (Hsu et al. 2016). We have observed that surrounding uterine muscular tissues do not prevent potassium iodine penetration and its even distribution within the sample. In addition, the uterine structures serve as a natural protection from the sample damaging and deformation during preparation and data acquisition by microCT. We also observed that the samples could be stored in Lugol solution for up to 1 year and then reanalyzed without noticeable damage of the samples and image quality losses. It has been shown previously that the iodine stained embryos dissected from the uterus but with intact deciduas prepared between E8.5–E10.5 can be imaged with the high-spatial resolution up to 3 µm/voxel by laboratory microCT (Hsu et al. 2016). Here, we demonstrated that the iodine contrast microCT imaging can be applied to analyze an entire litter of the developing embryos without dissection from the uterus. The whole-uterus iodine staining protocol can be applied also for the earliest embryos starting from E5.5–E7.5. While the protocol proposed by us for analysis of very early embryogenesis is advantageous because does not require substantial sample manipulations, the whole-uterus analysis of the mid-gestational period, when the embryos could be easily dissected and imaged individually, probably could be best suitable for the non-invasive morphometric analysis of the extra-embryonic developing tissues and for the volumetric visualization of the embryonic-extra-embryonic tissues cross talk during embryogenesis.

The microCT imaging is already broadly used for studies aimed to characterize embryonic lethality and to discover developmental phenotypes at middle to late-gestational and perinatal periods in mice in high-throughput manner by IMPC consortium (Dickinson et al. 2016). In order to unravel as many developmental phenotypes as possible in cost- and time-effective ways, novel protocols and methodologies to expand already existing phenotyping efforts are very advantageous. Here, we proposed and tested the application of the microCT imaging technology to study earliest embryonic lethality from peri- and immediate post-implantation to mid-gestation period. We developed two-step phenotyping scheme with the aim to define the interval of developmental arrest in mutant animals. The first step of the scheme defines the developmental window in which embryonic death occurs by genotyping of the individual embryos and by the low-resolution microCT imaging of the gravid uteri obtained from the crosses between heterozygous mutant animals. At the second step, the high-resolution X-ray imaging of the delayed embryos is applied to determine the precise point at which the arrest of embryonic development occurs and to characterize in details the possible morphological features that might lead to embryonic death.

In summary, our results suggest that whole-uterus microCT imaging analysis can be applied for studies with the goal to identify the genetic causes of early embryonic lethality in mutant animals and to investigate normal variation within the littermates. In addition, this methodology can be also efficient for the screening of the environmental and pharmacological components which can interfere with the normal embryonic development. The simplicity and robustness of the described methodology make it very suitable for the characterization of the gene function during embryogenesis for both small scale laboratories and high-throughput studies and can be easily implemented by any laboratory equipped by a microCT system.

## Materials and methods

### Ethics statement

All animals in this study were handled in accordance with the experimental protocols and animal care procedures, reviewed and approved by the Ethical and Scientific Commission of Veterinary Health and Welfare Department of the Italian Ministry of Health (protocol approval reference: 118/2012-B). The ethical and safety rules and guidelines for the use of animals in biomedical research provided by the Italian laws and regulations, in application of the relevant European Union’s directives (n. 86/609/EEC and 2010/63/EU).

### Animals

All animals in this study were produced and maintained on a C57BL/6N background by European Mouse Mutant Archive (EMMA) facility in Monterotondo, Roma, Italy. Animals were maintained in a temperature-controlled room at 21 ± 2 °C, on a 12-h light–dark cycle (lights on at 7 a.m. and off at 7 p.m.). After weaning, mice were housed by litter of the same sex, 3–5 per cage with food and water available ad libitum in a specific pathogen-free facility. For studies of embryonic development, time mating was set up around 5 p.m. and the plug were checked at 8 a.m. in the following morning. The plugged females were separated from the male and the pregnancy was scored as embryonic day 0.5.

*Tsen54* ^*tm1b*^/+ mice were produced by IKMC consortium at Monterotondo, Italy. *Tsen54* ^*tm1a(EUCOMM)Wtsi*^ ES cells (https://www.mousephenotype.org/data/genes/MGI:1923515) were injected in blastocysts and chimaeras were produced. Chimaeras transmitted targeted allele into progenies. Neomycin selection cassette flanked by *LoxP* sites was excised upon breeding with *Rosa26Cre* animals (Soriano 1999). With this breeding strategy, animals carrying *Tsen54* ^*tm1b*^ allele were generated. Genotyping of the *Tsen54* ^*tm1b*/+^ animals were performed with the following primers:*Tsen54 F*: TCTTTTTCCCACCCTTTGTG, *Tsen54 R*: AGCCGTGATTTGCTTTCTGT and *Frt-Rev*: CCTTCCTCCTACATAGTTGGCAGT; wild type allele produces a PCR product of 299 bp while *Tsen54* ^*tm1b*^ allele is 333 bp. in size (Supplementary Fig. 2). To study embryonic lethal phenotype, time mating was set up between heterozygous *Tsen54* ^*tm1b*/+^ animals.

### Samples preparation for microCT imaging

#### Paraffin embedding

Pregnant females were sacrificed by cervical dislocation at 8 a.m. of the indicated pregnancy day. The uteri with the embryos were dissected and washed with PBS and then fixed 4%PFA for 30 min. The gravid uteri were dehydrated by treatment with 50% ethanol for 30 min, 70% ethanol for 1 h, 95% ethanol for 30 min, all above listed treatments were performed in the cold room, then the samples were incubated in 100% ethanol at room temperature for 1 h and transferred into xylen for 30 min, xylen were replaced by a 1:1 ratio xylen/paraffin solution and samples were incubated at 56 °C for 15 min. Then the samples were transferred in paraffin and were incubated at 56 °C over night. Next morning, paraffin was replaced with the fresh paraffin for 1 h at 56 °C, transferred in the tissue embedding moulds and allowed to polymerize for about 1 h. Paraffin then was trimmed around the ages to fit into the holder of the microCT scanning machine.

#### Potassium iodine (Lugol) staining

After dissection gravid uteri were washed with PBS, fixed in 4%PFA for 30 min and transferred into 0.1N (v/v) Lugol solution (Sigma). Uteri were left in Lugol for two days at room temperature in a glass container in the dark. After, the samples were fitted in the narrow plastic column and placed in the microCT machine holder.

#### microCT imaging

The 3D raw data were acquired by Skyscan 1172G (Bruker, Kontich—Belgium) using a L7901-20 Microfocus X-ray Source (Hamamatsu). The source uses an X-ray tube with beryllium output window (150 µm thick), with a focal spot size of 7 µm (10W) or 5 µm (at 4W) able to work at a maximum tube voltage of 100 kV. The X-ray tube voltage range, which is adjustable from 20 to 100 kV, was set at 39 kV, while the X-ray tube current was set at 240 µA (9 W). In all presented experiments, the image acquisition was performed without filter. The samples were rotated 360° during the volume acquisition. For low-resolution image acquisition modality, a projection image at 1000 × 575 pixels was generated every 0.4° rotation and an average every 3 images; the high-resolution projection images were acquired at either 2000 × 1150 pixels or at 4000 × 2300 pixels which were generated at every 0.2° rotation at an average of every 3 images. Reconstructions of the acquired 2D images in volume images were performed using built-in NRecon Skyscan reconstruction software (Version:1.6.6.0; Bruker).The reconstructed tomographic datasets were stored as .BMP(8)-files (dim. 1000 × 1000). The 3D images were visualized using 3D Visualization Software CTvox v 2.5 (Bruker) to the volume rendering views and movies.

Preparation of the images for presentation was performed using ImageJ software. The images from “The Atlas of Mouse Development” were modified by refreshing brightness and contrast of the original images, using Photoshop application. In addition, the numbering on annotation is differ from the original images of the Atlas of Mouse Development. It has been modified to follow the numbering logic of our figures.

#### The embryonic volume measuring

The calculations were performed using Bruker microCT-Analyser Version 1.13 software. The embryo was manually defined as a region of interest (ROI) in every 2D section acquired by microC and separate data set containing only the embryo was created. The volume of the embryo was calculated using automatic thresholding to define embryonic tissues within this data set as the volume of interest (VOI).

## Electronic supplementary material

Below is the link to the electronic supplementary material.

MicroCT imaging of the murine conceptuses from E5.5–E7.5 treated with potassium iodine contrasting agent at 2.9 μm/voxel resolution. **A**, **B** High-resolution 2D virtual sections of volume reconstructed microCT images of murine E5.5 conceptus with the embryo at egg cylinder stage: sagittal (**A**) and transverse (B) sections. **C**, **D** 2D virtual sections of the microCT produced volume image at E6.5 days of development: sagittal (**C**); transverse (**D**) sections. **E**, **F** 2D virtual sections of microCT produced volume image at E7.5 days: sagittal (**F**); transverse (**F**) sections. Supplementary material 1 (PPTX 2843 KB)

Engineering of Tsen54 gene knockout in mice. **A** Schematic representation of the Tsen54^Tm1a(EUCOMM)Wtsi^ targeted allele in the ES cells. Targeted ES cell clone were obtained from EUCOMM consortium (http://www.mousephenotype.org/data/search/allele2?kw=%22Tsen54%20tm1a(EUCOMM)Wtsi%22). Left and right homology arms of targeting construct are indicated, reporter* LacZ* gene,* neo* cassette and exon6 of* Tsen54* gene flanked by* LoxP* sites are delineated. Probe for southern blot analysis of the correct integration is marked. **B** Southern blot analysis of genomic Hind III digested DNA obtained from targeted *Tsen54* ^Tm1a(EUCOMM)Wtsi^ ES cell clones. Wild type allele (Wt) produce 31.7 kb restriction fragment and correctly targeted clones revealed targeted allele (tg) of 14.3 kb. **C** Long Range PCR analysis of targeted clones. Correctly targeted clones produced PCR product of 6.3 kb with primers 1-(*Tsen54* FP: 5′-gccatccgccatccgccaactcctc 3′) and 2-(*LAR3*: 5′-cacaacgggttcttctgttagtcc-3′); 5.0 kb with primers 3- (*R2R*:5′ tctatagtcgcagtaggcgg-3′) and 4-(*Tsen54* RP: 5′-ctcttcagaagtccatcaactccatgatc-3′), demonstrating correct integration of both left and right homology arms of the targeting vector. **D** Schematic representation of *Tsen54* ^Tm1a(EUCOMM)Wtsi^ allele in mice. *Tsen54* ^Tm1b^ allele is produced from *Tsen54* ^Tm1(EUCOMM)Wtsi^ allele after Cre-mediated deletion of LoxP-flanked sequences following breeding with Gt(ROSA)26Sor<tm1(ACTB-cre,-EGFP)Ics mice and replacing exon6 of *Tsen54* gene with the *LacZ* reporter gene. The deletion was confirmed by the PCR with the following primers:1-(*E822*: 5′-aactggcagatgcacggttacgat-3′) and (*LoxP rev*:5′-actgatggcgagctcagaccataa-3′). Correct PCR product confirmed conversion of the *Tsen54* ^Tm1a(EUCOMM)Wtsi^ allele into knockout *Tsen54* ^Tm1b^ allele. Supplementary material 2 (PPTX 256 KB)

3D volume rendering of the paraffin embedded mouse conceptus at (E5.5) with the embryo at egg cylinder stage of development (1.4 μm/voxel resolution). Supplementary material 3 (AVI 483728 KB)

3D volume rendering of the paraffin embedded mouse conceptus (E6.5); embryo is at advanced egg cylinder stage of development (1.4 μm/voxel resolution). Supplementary material 4 (AVI 393725 KB)

3D volume reconstructed view of the embedded in paraffin mouse conceptus with the embryo at primitive streak stage (E7.5) at 1.4 μm/voxel resolution. Supplementary material 5 (AVI 420146 KB)

3D volume reconstructed view of the embedded in paraffin mouse conceptus with an advanced primitive streak stage,in the process of proamniotic canal closure (E7.75) at 1.4 μm/voxel resolution. Supplementary material 6 (AVI 413267 KB)

3D Volume reconstructed view of the paraffin embedded mouse conceptus staged as early headfold presomite stage embryo (E7.75) at 1.4 μm/voxel resolution. Supplementary material 7 (AVI 488020 KB)

3D volume reconstructed view of the potassium iodine stained E8.5 mouse conceptus with the embryo in the process of “turning” at 3.9 μm/voxel resolution. Supplementary material 8 (AVI 644646 KB)

3D volume reconstructed view of the potassium iodine stained E9.5 mouse conceptus with the embryo which has completed the process of “turning”at 3.9 μm/voxel resolution. Supplementary material 9 (AVI 435145 KB)

3D volume reconstructed view of potassium iodine stained E10.5 mouse conceptus sectioned in sagittal and transverse planes at 3.9 μm/voxel resolution. Supplementary material 10 (AVI 301305 KB)

3D volume reconstructed view of potassium iodine stained E11.5 mouse conceptus sectioned in sagittal and transverse planes at 3.9 μm/voxel resolution. Supplementary material 11 (AVI 299280 KB)

3D volume reconstructed view of potassium iodine stained E12.5 mouse conceptus sectioned in sagittal and transverse planes at 7.9 μm/voxel resolution. Supplementary material 12 (AVI 328772 KB)

## References

[CR1] Adams D (2013). Bloomsbury report on mouse embryo phenotyping: recommendations from the IMPC workshop on embryonic lethal screening. Dis Models Mech.

[CR2] Dhungel N, Hopper AK (2012). Beyond tRNA cleavage: novel essential function for yeast tRNA splicing endonuclease unrelated to tRNA processing. Genes Dev.

[CR3] Dickinson ME (2016). High-throughput discovery of novel developmental phenotypes. Nature.

[CR4] Downs KM, Davies T (1993). Staging of gastrulating mouse embryos by morphological landmarks in the dissecting microscope. Development.

[CR5] Gregg CL, Butcher JT (2016). Comparative analysis of metallic nanoparticles as exogenous soft tissue contrast for live in vivo micro-computed tomography imaging of avian embryonic morphogenesis. Dev Dyn.

[CR6] Gregg CL (2015). Micro/nano-computed tomography technology for quantitative dynamic, multi-scale imaging of morphogenesis. Methods Mol Biol.

[CR7] Hsu CW (2016). Three-dimensional microCT imaging of mouse development from early post-implantation to early postnatal stages. Dev Biol Nov.

[CR8] Ichikawa T (2014). Live imaging and quantitative analysis of gastrulation in mouse embryos using light-sheet microscopy and 3D tracking tools. Nat Protoc.

[CR9] Johnson JT (2006). Virtual histology of transgenic mouse embryos for high-throughput phenotyping. PLoS Genet.

[CR10] Kauffman MH (1999). The atlas of mouse development.

[CR11] Kotorashvili A (2012). Effective DNA/RNA co-extraction for analysis of microRNAs, mRNAs, and genomic DNA from formalin-fixed paraffin-embedded specimens. PLoS ONE.

[CR12] Martelotto LG (2017). Whole-genome single-cell copy number profiling from formalin-fixed paraffin-embedded samples. Nat Med.

[CR13] Metscher BD (2009). MicroCT for developmental biology: a versatile tool for high-contrast 3D imaging at histological resolutions. Dev Dyn.

[CR14] Metscher BD (2009). MicroCT for comparative morphology: simple staining methods allow high-contrast 3D imaging of diverse non-mineralized animal tissues. BMC Physiol.

[CR15] Mohun T (2013). Deciphering the Mechanisms of Developmental Disorders (DMDD): a new programme for phenotyping embryonic lethal mice. Dis Model Mech.

[CR16] Neues F, Epple M (2008). X-ray microcomputer tomography for the study of biomineralized endo- and exoskeletons of animals. Chem Rev.

[CR17] Norris FC (2013). A coming of age: advanced imaging technologies for characterizing the developing mouse. Trends Genet.

[CR18] Papaioannou VE, Behringer RR (2012). Early embryonic lethality in genetically engineered mice: diagnosis and phenotypic analysis. Vet Pathol Jan.

[CR19] Rands GF (1986). Size regulation in the mouse embryo. I. The development of quadruple aggregates. J Embryol Exp Morphol.

[CR20] Schambach SJ (2010). Application of micro-CT in small animal imaging. Methods.

[CR21] Sharpe J (2002). Optical projection tomography as a tool for 3D microscopy and gene expression studies. Science.

[CR22] Soriano P (1999). Generalized lacZ expression with the ROSA26 Cre reporter strain. Nat Genet.

[CR23] Udan RS (2014). Quantitative imaging of cell dynamics in mouse embryos using light-sheet microscopy. Dev Nov.

[CR24] Weninger WJ (2006). High-resolution episcopic microscopy: a rapid technique for high detailed 3D analysis of gene activity in the context of tissue architecture and morphology. Anat Embryol.

[CR25] Weninger WJ (2014). Phenotyping structural abnormalities in mouse embryos using high-resolution episcopic microscopy. Dis Model Mech.

[CR26] White JK (2013). Genome-wide generation and systematic phenotyping of knockout mice reveals new roles for many genes. Cell.

[CR27] Wilson R (2016). DMDD Project.Deciphering the mechanisms of developmental disorders: phenotype analysis of embryos from mutant mouse lines. Nucleic Acids Res.

[CR28] Wong MD (2012). A novel 3D mouse embryo atlas based on micro-CT. Development.

[CR29] Wong MD (2013). Structural stabilization of tissue for embryo phenotyping using micro-CT with iodine staining. PLoS ONE.

[CR30] Wong MD (2015). 4D atlas of the mouse embryo for precise morphological staging. Development.

