# Supplementary figures and images for: Three-dimensional microCT imaging of murine embryonic development from immediate post-implantation to organogenesis: application for phenotyping analysis of early embryonic lethality in mutant animals
Source: Mamm Genome. 2017 Nov 23;29(3):245–59. doi: 10.1007/s00335-017-9723-6 (PMC5887010; doi:10.1007/s00335-017-9723-6)

## Slide 1
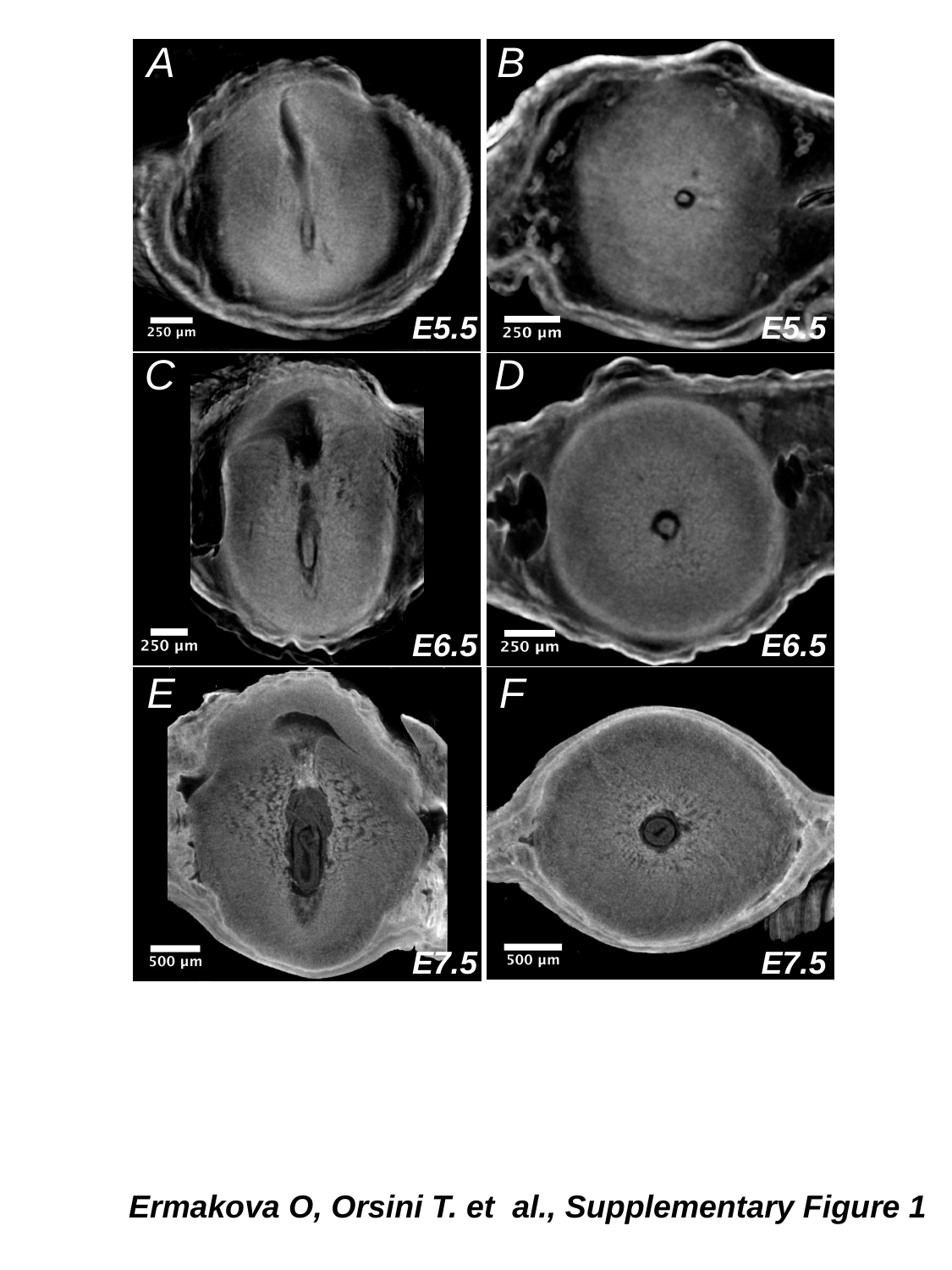

A
B
E5.5
E5.5
C
D
E6.5
E6.5
E
F
E7.5
E7.5
 Ermakova O, Orsini T. et al., Supplementary Figure 1

Supplement: Supplementary file 1 — MicroCT imaging of the murine conceptuses from E5.5–E7.5 treated with potassium iodine contrasting agent at 2.9 μm/voxel resolution. A, B High-resolution 2D virtual sections of volume reconstructed microCT images of murine E5.5 conceptus with the embryo at egg cylinder stage: sagittal (A) and transverse (B) sections. C, D 2D virtual sections of the microCT produced volume image at E6.5 days of development: sagittal (C); transverse (D) sections. E, F 2D virtual sections of microCT produced volume image at E7.5 days: sagittal (F); transverse (F) sections. Supplementary material 1 (PPTX 2843 KB) [file 335_2017_9723_MOESM1_ESM.pptx]
