# Supplementary material for: Three-dimensional microCT imaging of murine embryonic development from immediate post-implantation to organogenesis: application for phenotyping analysis of early embryonic lethality in mutant animals
Source: Mamm Genome. 2017 Nov 23;29(3):245–59. doi: 10.1007/s00335-017-9723-6 (PMC5887010; doi:10.1007/s00335-017-9723-6)
Supplement: Supplementary file 2 — Engineering of Tsen54 gene knockout in mice. A Schematic representation of the Tsen54Tm1a(EUCOMM)Wtsi targeted allele in the ES cells. Targeted ES cell clone were obtained from EUCOMM consortium (http://www.mousephenotype.org/data/search/allele2?kw=%22Tsen54%20tm1a(EUCOMM)Wtsi%22). Left and right homology arms of targeting construct are indicated, reporter LacZ gene, neo cassette and exon6 of Tsen54 gene flanked by LoxP sites are delineated. Probe for southern blot analysis of the correct integration is marked. B Southern blot analysis of genomic Hind III digested DNA obtained from targeted Tsen54 Tm1a(EUCOMM)Wtsi ES cell clones. Wild type allele (Wt) produce 31.7 kb restriction fragment and correctly targeted clones revealed targeted allele (tg) of 14.3 kb. C Long Range PCR analysis of targeted clones. Correctly targeted clones produced PCR product of 6.3 kb with primers 1-(Tsen54 FP: 5′-gccatccgccatccgccaactcctc 3′) and 2-(LAR3: 5′-cacaacgggttcttctgttagtcc-3′); 5.0 kb with primers 3- (R2R:5′ tctatagtcgcagtaggcgg-3′) and 4-(Tsen54 RP: 5′-ctcttcagaagtccatcaactccatgatc-3′), demonstrating correct integration of both left and right homology arms of the targeting vector. D Schematic representation of Tsen54 Tm1a(EUCOMM)Wtsi allele in mice. Tsen54 Tm1b allele is produced from Tsen54 Tm1(EUCOMM)Wtsi allele after Cre-mediated deletion of LoxP-flanked sequences following breeding with Gt(ROSA)26Sor<tm1(ACTB-cre,-EGFP)Ics mice and replacing exon6 of Tsen54 gene with the LacZ reporter gene. The deletion was confirmed by the PCR with the following primers:1-(E822: 5′-aactggcagatgcacggttacgat-3′) and (LoxP rev:5′-actgatggcgagctcagaccataa-3′). Correct PCR product confirmed conversion of the Tsen54 Tm1a(EUCOMM)Wtsi allele into knockout Tsen54 Tm1b allele. Supplementary material 2 (PPTX 256 KB) [file 335_2017_9723_MOESM2_ESM.pptx]

## Slide 1
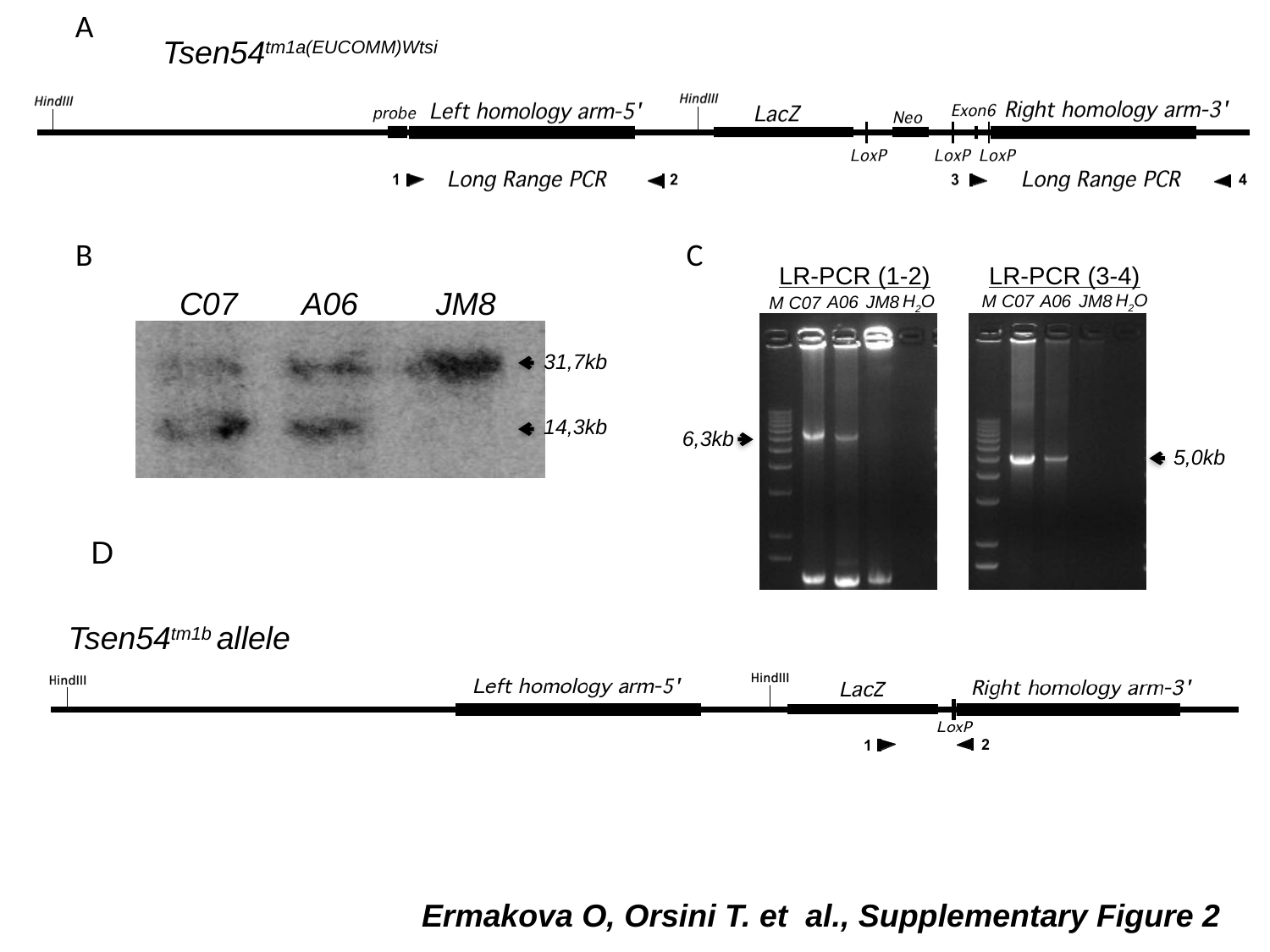

A
Tsen54tm1a(EUCOMM)Wtsi
B
C
LR-PCR (1-2)
LR-PCR (3-4)
C07
A06
JM8
H2O
A06
JM8
H2O
M
C07
A06
JM8
M
C07
31,7kb
14,3kb
6,3kb
5,0kb
D
Tsen54tm1b allele
 Ermakova O, Orsini T. et al., Supplementary Figure 2
